# Association of Angiotensin Converting Enzyme Insertion-Deletion Polymorphism with Hypertension in Emiratis with Type 2 Diabetes Mellitus and Its Interaction with Obesity Status

**DOI:** 10.1155/2015/536041

**Published:** 2015-09-29

**Authors:** Habiba Alsafar, Ahmed Hassoun, Shaikha Almazrouei, Wala Kamal, Mustafa Almaini, Unini Odama, Naushad Rais

**Affiliations:** ^1^Department of Biomedical Engineering, Khalifa University of Science, Technology & Research, P.O. Box 127788, Abu Dhabi, UAE; ^2^Dubai Diabetes Centre, Dubai Health Authority, Dubai, UAE; ^3^School of Life Sciences, Manipal University, P.O. Box 345050, Dubai, UAE; ^4^Immunology Clinics, Mafraq Hospital, Abu Dhabi, UAE; ^5^Landmark Nephrology and Hypertension Clinic, Talladega, AL, USA

## Abstract

The association of Angiotensin Converting Enzyme (*ACE*) insertion-deletion (I/D) polymorphism with Type 2 Diabetes Mellitus (T2DM) and hypertension has been extensively studied throughout various ethnic populations but largely with inconsistent findings. We investigated these associations in Emirati population and their interaction with obesity status. Saliva samples were collected from a total of 564 Emiratis (277 T2DM and 297 healthy). DNA was extracted and the samples were genotyped for *ACE* I/D polymorphism by a PCR based method followed by gel electrophoresis. Upon evaluation of the *ACE* I/D polymorphism amongst all T2DM, hypertensive patients, and respective controls regardless of obesity status, *ACE* DD genotype was not found to be associated with either T2DM [odds ratio (OR) = 1.34, *p* = 0.086] or hypertension [odd ratio (OR) = 1.02, *p* = 0.93]. When the genetic variants amongst the nonobese and obese population were analyzed separately, the risk genotype *ACE* DD conferred significantly increased risk of hypertension in nonobese population [odds ratio (OR) = 1.80, *p* = 0.02] but was found to be protective against the hypertension in the obese group ((OR) = 0.54, *p* = 0.01). However, there was no effect of obesity status on the association of *ACE* genotypes with T2DM. The risk of hypertension associated with *ACE* DD is modulated by obesity status and hence future genetic association studies should take obesity into account for the interpretation of data. We also confirmed that *ACE* I/D polymorphism is not associated with T2DM risk in Emirati population.

## 1. Introduction

The clustering of Type 2 Diabetes Mellitus (T2DM), hypertension, and obesity along with dyslipidemia is termed metabolic syndrome which is prevalent in populations of modernized nations. Hypertension is common and is a major risk factor for cardiovascular (CVD) and coronary heart disease, particularly when associated with diabetes [1, 2]. The rising prevalence has been attributed to sedentary lifestyle related factors; however, genetic predisposition may play a vital role in the etiology and manifestation of the disease [3–5]. Recently, an ample amount of studies has focused on genetic variants in Renin-Angiotensin-Aldosterone System (RAAS) in association with the components of metabolic syndrome, especially hypertension and T2DM [6–8]. The association between insulin resistance, hyperinsulinemia, and the RAAS independent of plasma potassium and cortisol levels has been shown [9] and has been implicated in the development of cardiovascular and renal diseases in T2DM [10]. Most of these studies have focused on Angiotensin Converting Enzyme (*ACE*) that converts less active angiotensin I to more active angiotensin II which mediates a variety of cellular functions in different tissues.

The* ACE *gene located on chromosome 17q23 is highly polymorphic with an insertion/deletion of a 287-bp element within intron 16 being the most common and most studied polymorphism which results in three genotypes (D/D, I/D, and I/I) [11, 12]. The D allele particularly in homozygous (DD) state is associated with high level of circulating* ACE* resulting in an increased activity of angiotensin II which may predispose the individual to a variety of disorders including T2DM and hypertension. But a survey of literature showed inconsistencies among the studies on the* ACE* gene polymorphisms disease outcome. The frequency of D allele varies widely from 0.31–0.35 in Thai and Malaysian population [13, 14] to 0.75 in Arabs [15, 16]. According to International Diabetes Federation (November 14, 2014), the Emirati population is also amongst the populations with one of the highest T2DM prevalence, ranked 16th world wide with 19% of the UAE population living with T2DM; hence, an association between* ACE* D allele and T2DM could be expected. The association of* ACE* I/D polymorphism with T2DM risk and related renal and cardiovascular complications has been extensively studied throughout all the major ethnic groups but with highly inconsistent findings. Whereas some have found that the D allele is more common in T2DM and related complications in Tunisian [16], Indian [17], and Iranian populations [18], others have demonstrated no association of either allele with T2DM or related cardiovascular and renal disease in Malay and Indonesians [19–21]. The association between these polymorphisms and the* ACE* gene is similarly discrepant between its association with hypertension in the Japanese [22] and its nonassociation in the Chinese [23]. These differences are mostly due to multifactorial or polygenic disorders, as evidenced by the varying disease outcomes modulated by the gene-gene or gene-environment interactions. The genetic susceptibility to T2DM has been found to be modulated by obesity status [24]. Insulin resistance related genetic variants were associated with T2DM only in obese population while insulin secretory variants conferred a greater T2DM risk only in nonobese individuals, suggesting potential interaction of these variants with obesity status in T2DM occurrence [24]. Recently, Fujii et al. [25] reported that copresence of abdominal obesity and a high level of high-sensitivity C-reactive protein (hsCRP) are associated with a high risk for new-onset hypertension in the general Japanese population. Previous genome-wide association studies on diabetes, obesity, and hypertension have identified many genetic markers that have significant associations [26–28]. However, most studies reported only a marginal effect of the Single Nucleotide Polymorphism (SNP) on T2DM disease, and very few reported interaction effects [29], either among genes or between genes, and environmental factors such as obesity status. Some studies were conducted to address the gene-gene interactions in T2DM, but nothing significant was discovered [30]. Hence, we hypothesized that genetic associations of the* ACE* gene with T2DM or hypertension may depend on the obesity status. Our previous study failed to demonstrate any association between the* ACE* I/D polymorphism and T2DM [31]; therefore, our first objective was to further investigate the relationship between the* ACE* gene polymorphisms with T2DM risk again, but this time in a much larger and more clinically and biochemically characterized Emirati population, using an improved genotyping method. Second objective was to investigate the association between* ACE* I/D polymorphism and hypertension risk. These objectives were achieved by determining and comparing the genotypic frequencies of* ACE* gene I/D polymorphism in obese and nonobese T2DM and hypertension patients.

## 2. Materials and Methods

### 2.1. Subjects and Sample Collection

This study was conducted on five hundred and sixty four (*n* = 564) unrelated Emiratis who were identified during their routine visit to an endocrinology clinic in Dubai and Abu Dhabi, United Arab Emirates, between the period of June 2012 and Dec 2013 and agreed to take part in this study by signing informed consents that had been IRB approved. The case group of 277 Type 2 Diabetes Mellitus patients (180 hypertensive and 97 normotensive) had a mean age of 58 ± 12 years and consisted of 58.5% females. The control group consisted of 297 nondiabetics individuals (55 hypertensive and 236 normotensive) who had mean age of 45 ± 16 years and consisted of 49.2% females. All participants gave their informed consent in writing. There were several inclusion and exclusion criteria used in this study. Inclusion criteria include being UAE national, diagnosed with T2DM, healthy individuals, able to give consent, and above 18 years old. Exclusion criteria include being non-UAE national, pregnant female, not be able to consent, and less than 18 years old. This study was approved by the ethics committees of the Dubai Health Authority and Mafraq Hospital Research in the United Arab Emirates.

After providing informed consent, a registered nurse asked each participant to deliver 1 mL of saliva into an Oragene kit OGR-500 (DNA Genotek, Ottwa, Canada) for DNA samples. In addition, clinical assessments and lifestyle questionnaire were completed at the clinic to study any correlation between lifestyle variables and genetic variation. An individual was classified as T2DM if the subject (1) was diagnosed with T2DM by a qualified physician, (2) was on a prescribed drug treatment regimen for T2DM, and (3) returned biochemical test results of a fasting plasma glucose level of at least 126 mg/dL based on the criteria outlined by the World Health Organization (WHO) consultation group report [32]. The individuals with BMI more than 30 were considered obese and those with a BMI less than 30 were grouped into the nonobese population. According to JNC 8 classification, all the individuals with blood pressure more than 140/90 mmHg were considered hypertensive [33]. The saliva samples were preserved for DNA extraction and genotyping. The genomic DNA was extracted from saliva using prepITTM·L2P (DNA Genotek, Ottwa, Canada) in accordance with the manufacturer's instructions.

### 2.2. PCR Based Genotyping for* ACE* I/D Polymorphism

The genotyping for the insertion in intron 16 of* ACE* gene was performed by two subsequent PCR reactions using the primers and PCR cycling conditions described by Lovati et al. [34]. In brief, the first PCR was done in a 50 *μ*L reaction mixture containing 1x PCR Taq buffer (Applied Biosystems), 3 mM MgCl_2_, 0.2 mM of dNTPs (Fermentas), 1 *μ*M of each reverse and forward primer (Sigma-Aldrich), 1.5 U Taq DNA polymerase (Applied Biosystems), and approximately 100 ng DNA. After initial denaturation at 95°C for 5 min, the thermocycling procedure consisted of denaturation at 95°C for 30 s, annealing at 59°C for 45 s, and extension at 72°C for 30 s, repeated for 35 cycles and followed by a final extension at 72°C for 10 min. The* ACE* DD genotypes were confirmed by subjecting to a second independent PCR using the primers that recognize an insertion-specific sequence. The reaction mixture was prepared as described for the first PCR and the thermal cycling conditions were as follows: 35 cycles of 94°C for 30 sec, 67°C for 45 sec, and 72°C for 120 sec. All PCR reactions took place in Veriti 96-well thermocyclers (Applied Biosystems). PCR products were separated on 2% agarose gels and visualized by ethidium bromide staining under UV transilluminator.

The first PCR reaction yields a 190 bp fragment in the absence of insertion (D allele) and a 490 bp fragment in the presence of insertion (I allele). The samples that demonstrated only the D allele (DD genotypes) were further analyzed as described above. In case of mistyping, a 335 bp product was identified as a result of second PCR in those samples that hence were considered heterozygotes (ID genotypes), while no band was detected when the DD genotyping was correct.

### 2.3. Statistical Analysis

Data from normally distributed parameters are presented as means ± SD. Differences in the genotype and allele frequencies between the different groups were analyzed using the Fisher exact test and odds ratios with 95% CIs. The genotype frequencies were tested for the Hardy-Weinberg equilibrium using a *χ* ^2^ test. Only one gene transmission model, that is, recessive effect (DD versus ID + II) of the D allele, was tested. The association was evaluated by Chi square test followed by logistic regression. A *p* value of <0.05 was considered for statistical significance. The interaction of* ACE* genotypes (II, ID, and DD), age, BMI, and gender and the relationship of DD with either T2DM or hypertension were also evaluated with logistic regression using the two by four tables. Differences in biochemical parameters between different groups and between the genotypes were examined using Student's *t*-test.

## 3. Results

The demographic characteristics and clinical and biochemical parameters of T2DM patients and healthy control patients are described in Table 1. The mean age and male/female ratios significantly differed in the affected individuals and healthy controls. The cases were significantly older than the controls (58 years ± 12 versus 45 years ± 16, *p* < 0.001); their mean BMI, lipid profile, and blood pressure levels were also significantly different (Table 1). However, no significant differences were observed in these parameters between different* ACE* genotypes (DD versus II + ID) except that HBA1c was slightly higher in the subjects with DD genotypes (7.36 versus 6.92 mmol/L, *p* = 0.028) (Table 2).

### 3.1. Allele and Genotype Frequency Distribution of* ACE* I/D Polymorphism and Association with T2DM

#### 3.1.1. All Nondiabetics and T2DM Individuals regardless of Obesity Status

The frequencies of* ACE* II genotype were significantly higher in controls than the T2DM cases (10.8% versus 1.4%, *p* = 0.001).* ACE* DD and ID genotype frequencies were 44.8 and 53.8%, respectively, in T2DM group and 37.7 and 51.5%, respectively, in controls regardless of obesity status (Table 3). The frequency of D allele was 0.72 (72%) in T2DM patients, significantly higher than 0.63 (63%) in healthy individuals (*p* = 0.0037), but the carriers of DD genotype were not significantly associated with T2DM risk [OR = 1.34 (0.96–1.87), *p* = 0.086] (Table 3). The genotype distributions were in Hardey-Weinberg equilibrium in the nondiabetic control group (*p* = 0.054).

#### 3.1.2. *ACE* I/D and T2DM Association in Obese and Nonobese Population

Allele and genotype frequency distribution for* ACE* polymorphism analyzed separately for obese and nonobese individuals for T2DM showed no significant differences when compared with the group as a whole without stratifying by obesity status. There were no significant differences in DD frequency between cases and control in the nonobese or obese population (Table 4).

### 3.2. Allele and Genotype Frequency Distribution of* ACE* I/D Polymorphism and Association with Hypertension

#### 3.2.1. Regardless of Obesity Status

The frequencies of* ACE* DD genotypes were 41.7 and 40.8%, respectively, in hypertensive cases and normotensive controls regardless of obesity and T2DM status (Table 3). The differences were not statistically significant (*p* = 0.86) indicating no association of* ACE* DD genotypes with hypertension. However, the frequency of* ACE* II genotype was significantly higher in control group (10.3 versus 0.9%, *p* < 0.001) (Table 3). The genotype distributions were in Hardey-Weinberg equilibrium in the normotensive control group (*p* = 0.14).

#### 3.2.2. Hypertension and* ACE* I/D Association Based on Obesity Status

When we assessed the* ACE* I/D variant amongst the obese and nonobese population separately, the frequency of DD genotype was found to be significantly higher in the normotensive group (30 versus 44%, *p* = 0.02) than in the hypertensive obese individuals but its frequency was higher in the hypertensive nonobese individuals (*p* = 0.03) (Table 4) indicating that the association of* ACE* I/D polymorphism with hypertension is dependent on obesity status. The risk genotype DD conferred significantly greater hypertension risk (OR 1.80 (95% CI 1.0–2.99), *p* = 0.02) in the nonobese group (Table 4).

## 4. Discussion

The RAS plays a fundamental role in controlling blood pressure and each component of it has been shown to be involved in the pathogenesis of primary hypertension. This is best illustrated by the fact that* ACE* and other RAS inhibitors may reduce the risk from CVDs. In addition, the RAS may also affect other aspects of the pathogenesis of the metabolic syndrome such as atherosclerosis and insulin resistance [33, 34], both of which are reported to be reduced by* ACE* inhibitors. It has been suggested that activation of RAS through insulin resistance may cause the development of dyslipidemia and diabetes which was supported by finding that* ACE* inhibitors were able to reduce the lipolysis induced by insulin resistance in adipose tissues of centrally obese hypertensive individuals [35]. In addition, I allele of* ACE* I/D polymorphism was found to be associated with increased indexes of insulin resistance in nondiabetic Caucasians and Africans [36, 37]. So we hypothesized that T2DM may be a result of complex interplay among polymorphism in* ACE* genes, hypertension, and obesity, even though in our previous study we were not able to correlate the* ACE* I/D polymorphism alone with the T2DM risk [31]. However, we had certain limitations in that study related to sample size, lack of biochemical and clinical characterization of patients, and* ACE* I/D genotyping technique. We planned this study with a large and a very well defined population to evaluate the association of* ACE* polymorphism with T2DM and hypertension in the presence and absence of obesity.

The results suggested that there was no association between* ACE* DD genotype and T2DM. Although we observed a significantly high frequency of II genotype in the nondiabetic individuals, it did not reach statistical significance possibly because of very small numbers of II genotypes in each group. The genotype and allele frequencies were in concordance with previous reports in Emirati and other Arab populations [15, 16, 38]. The association of DD genotype with increased T2DM risk was also reported in Tunisians [16] and Iranians [18]. The variants of genes like* PPARG*,* ADIPOQ*, and* ENPP1* which act at the level of insulin resistance have been shown to increase the risk of T2DM only in obese populations [39–41] and hence it has been suggested that T2DM risk contribution of gene polymorphism may be modulated by obesity status. While obesity has been strongly associated with increased insulin resistance [42] and* ACE* being one of the factors associated with insulin resistance as discussed elsewhere, variants like* ACE* D allele may be expected to more significantly modulate T2DM risk in obese subjects. In our previous study [31], the data about obesity status was not available and therefore it is possible that the participants in our previous study were mainly nonobese, such that we did not find an association of* ACE* I/D polymorphism with T2DM. Moreover, in the present study, the DD genotypes were confirmed by an additional confirmatory PCR that resulted in more accurate distribution of* ACE* genotypes. In the metabolic syndrome, hypertension is clustered with diabetes [43], the development of which in the presence or absence of diabetes may result from a different kind of interaction between RAS gene polymorphisms and obesity. It was therefore important to assess the association of* ACE* I/D polymorphism with hypertension in diabetic and nondiabetic groups separately. Our study did not find any association of* ACE* I/D polymorphism with hypertension in combined diabetic and nondiabetic hypertensive patients versus nonhypertensive controls (Tables 3 and 4). However, when the association was analyzed based on obesity status, we found significantly higher* ACE* DD genotype frequency in the hypertensive groups and an increased risk associated with it in the nonobese hypertensive group but not in the obese hypertensive group. Interestingly, DD genotype was found to be protective against hypertension in the individuals with BMI more than 30.

This novel finding suggests that hypertension risk, as conferred by* ACE* I/D polymorphism, may also be modified by the presence or absence of obesity. Our results illustrate the importance of obesity in genetic association studies. We also provide important clues that might help explain part of the difficulties encountered in replication studies of this kind resulting in inconsistent findings. Our study also highlights obesity as an important modulator of* ACE* polymorphism, suggesting that future genetic association studies for hypertension should take obesity status into account when interpreting data.

The limitations of our study include the smaller sample size in the obese and nonobese subgroups created to study the association of* ACE* DD genotype with T2DM and hypertension. Residual confounding by the severity of diabetes might have come into play because patients with DD had a higher hemoglobin A1c (HbA1c) (Table 2). However, we performed accurate genotyping by confirmatory PCR and because the genotype distributions were in Hardy-Weinberg equilibrium in the control groups, the possibility of selection bias was also reduced.

In summary, in our study, we did not find* ACE* I/D polymorphism to be related to T2DM in the Emirati population. Notably, we found that the significant association between* ACE* DD genotype and hypertension was obesity status dependent. Therefore, we highlight the need for future genetic association studies for hypertension to consider obesity an important variable when analyzing results. The future will rely on more gene based modulation of disease and gene directed therapy; consequently, the importance of understanding the various modulators in chronic disease processes like hypertension and diabetes cannot be overemphasized and will increasingly rely on genetic studies with large sample sizes.

## Figures and Tables

**Table 1:** Demographic, clinical, and biochemical characteristics of T2DM patients and healthy subjects in the studied population.

Population demographics			
	T2DM cases *n* = 277	Nondiabetic controls *n* = 297	*p* ^*∗*^
Age (years)	58 ± 12	45 ± 16	<0.0001
Male : female ratio	115 : 162	151 : 146	0.02
Number of hypertensive	180	55	
Number of Normotensive	97	236	
BMI	31.93 ± 6.3	28.57 ± 6.23	<0.0001
Fasting glucose (mmol/L)	8.99 ± 7.23	5.63 ± 7.23	<0.0001
Systolic blood pressure	129.97 ± 17.24	122.50 ± 15.65	<0.0001
Diastolic blood pressure	70.0 ± 11.0	71.35 ± 11.45	0.6197
HBA1c (mmol/L)	7.6 ± 1.6	5.59 ± 0.56	<0.0001

Data shown is mean ± SD unless specified otherwise, ^*∗*^Student's *t*-test.

**Table 2:** Clinical and biochemical characteristics according to *ACE* genotypes in all the subjects.

Genotypes	HBA1c (mmol/L)	BMI (Kg/m^2^)	SBP (mmHg)	DBP (mmHg)
DD	7.36 ± 1.81	31.0 ± 6.70	127.1 ± 17.3	69.88 ± 10.76
II + ID	6.92 ± 1.57	30.9 ± 6.30	126.7 ± 16.8	71.36 ± 11.90
*p* ^*∗*^	0.028	0.924	0.830	0.205

Data shown is mean ± SD unless specified otherwise, ^*∗*^Student's *t*-test.

**Table 3:** Allele and genotype frequency distribution of *ACE* I/D polymorphisms in T2DM and hypertension cases and controls regardless of obesity status.

Frequency distribution						
	T2DM			Hypertension		
	Cases (*N* = 277)	Controls (*N* = 297)	*p* ^*∗*^	Cases (*N* = 235)	Controls (*N* = 333)	*p* ^*∗*^
Genotypes						
DD	124 (44.8%)	112 (37.7%)	0.09^†^ <0.0001^‡^	98 (41.7%)	136 (40.8%)	0.86^†^ <0.0001^‡^
ID	149 (53.8%)	153 (51.5%)		135 (57.4%)	163 (48.9%)	
II	4 (1.4%)	32 (10.8)		2 (0.9%)	34 (10.3%)	
Allele frequencies						
D	0.72	0.63	0.0037	0.70	0.65	0.07
I	0.28	0.37		0.30	0.35	

Data shown are numbers and percentages in parentheses unless specified otherwise, ^*∗*^Fisher exact test. ^‡^II versus ID + DD, ^†^DD versus ID + II.

**Table 4:** Evaluation of T2DM and hypertension risk association with *ACE* DD genotype by obesity status in case-control study.

Association based on obesity status							
	Obesity status	*ACE* genotype	Groups			Logistic regression	
			Cases	Control	*p* ^*∗*^	OR (95% CI)	*p*
T2DM	All together	DD	124	112	0.09	1.34 (0.96–1.87)	0.086
		ID + II	153	185			
	Obese (BMI > 30)	DD	66	38	0.30	1.34 (0.81–2.23)	0.26
		ID + II	88	68			
	Nonobese (BMI < 30)	DD	53	68	0.18	1.41 (0.88–2.28)	0.15
		ID + II	59	107			

Hypertension	All together	DD	98	136	0.86	1.02 (0.72–1.42)	0.93
		ID + II	137	197			
	Obese (BMI > 30)	DD	50	53	0.02	0.54 (0.33–0.89)	0.01
		ID + II	116	67			
	Nonobese (BMI < 30)	DD	46	75	0.03	1.80 (1.08–2.99)	0.02
		ID + II	41	120			

Data shown are numbers unless specified otherwise, ^*∗*^Fisher exact test.

All together means obese + nonobese and others about whom information on obesity status was not available.
